# Fgf-dependent otic induction requires competence provided by Foxi1 and Dlx3b

**DOI:** 10.1186/1471-213X-7-5

**Published:** 2007-01-19

**Authors:** Stefan Hans, Joe Christison, Dong Liu, Monte Westerfield

**Affiliations:** 1 Institute of Neuroscience, University of Oregon, Eugene, OR 97403, USA; 2 Biotechnology Center and Center of Regenerative Therapies, University of Technology, Dresden, Germany

## Abstract

**Background:**

The inner ear arises from a specialized set of cells, the otic placode, that forms at the lateral edge of the neural plate adjacent to the hindbrain. Previous studies indicated that fibroblast growth factors (Fgfs) are required for otic induction; in zebrafish, loss of both Fgf3 and Fgf8 results in total ablation of otic tissue. Furthermore, gain-of-function studies suggested that Fgf signaling is not only necessary but also sufficient for otic induction, although the amount of induced ectopic otic tissue reported after misexpression of *fgf3 *or *fgf8 *varies among different studies. We previously suggested that Foxi1 and Dlx3b may provide competence to form the ear because loss of both *foxi*1 and *dlx3b *results in ablation of all otic tissue even in the presence of a fully functional Fgf signaling pathway.

**Results:**

Using a transgenic line that allows us to misexpress *fgf8 *under the control of the zebrafish temperature-inducible *hsp70 *promoter, we readdressed the role of Fgf signaling and otic competence during placode induction. We find that misexpression of *fgf8 *fails to induce formation of ectopic otic vesicles outside of the endogenous ear field and has different consequences depending upon the developmental stage. Overexpression of *fgf8 *from 1-cell to midgastrula stages leads to formation of no or small otic vesicles, respectively. Overexpression of *fgf8 *at these stages never leads to ectopic expression of *foxi1 *or *dlx3b*, contrary to previous studies that indicated that *foxi1 *is activated by Fgf signaling. Consistent with our results we find that pharmacological inhibition of Fgf signaling has no effect on *foxi1 *or *dlx3b *expression, but instead, Bmp signaling activates *foxi1*, directly and *dlx3b*, indirectly. In contrast to early activation of *fgf8*, *fgf8 *overexpression at the end of gastrulation, when otic induction begins, leads to much larger otic vesicles. We further show that application of a low dose of retinoic acid that does not perturb patterning of the anterior neural plate leads to expansion of *foxi1 *and to a massive Fgf-dependent otic induction.

**Conclusion:**

These results provide further support for the hypothesis that Foxi1 and Dlx3b provide competence for cells to respond to Fgf and form an otic placode.

## Background

The vertebrate inner ear provides auditory and vestibular functions and develops from the otic placode, a transient thickening of head ectoderm adjacent to the developing hindbrain. In zebrafish the otic placode cavitates to form the otic vesicle also known as the otocyst, an epithelial structure with sharply defined borders, which later gives rise to the inner ear including its neurons and structural elements [1-4].

Studies in various species suggest that otic placode formation is a multi-step process. Cranial placodes, including the otic placode, arise from a common region, the preplacodal domain that extends around the anterior neural plate border. Formation of the preplacodal domain represents the earliest stage of placode induction and is regulated by multiple signaling pathways including Bmp, Wnt and Fgf [5-7]. Subsequently, Fgf signals from the adjacent hindbrain and underlying mesoderm induce cells within the preplacodal domain to form the otic placode (reviewed in [3,8-10]). In zebrafish, Fgf3 and Fgf8 have been implicated as having overlapping functions in otic placode induction [11-13]. Both genes are expressed in the future hindbrain by late gastrula stages, and *fgf3 *is also expressed at this stage in the underlying mesendoderm. Loss of either *fgf3 *or *fgf8 *leads to a reduction in ear size and loss of both *fgf3 *and *fgf8 *together results in near or total ablation of otic tissue. In mouse, Fgf3 and Fgf10 act as redundant signals during otic induction [14,15]. Fgf3 is expressed in the hindbrain region that abuts the preotic domain, whereas Fgf10 is expressed in the mesoderm beneath it; loss of both Fgf3 and Fgf10 results in complete ablation of otic development [14,15]. Furthermore, Fgf8 has been shown to play a critical role upstream of the FGF signaling cascade required for otic induction in this species [16]. In chick, Fgf3, Fgf8 and Fgf19 and in amphibians, Fgf2 and Fgf3 have been implicated in otic induction [16-20].

Misexpression studies indicate that Fgf signaling is not only necessary but also sufficient for otic induction [11,13,21-25]. However, the region where ectopic otic structures formed was inconsistent in these studies, ranging from limited induction in the region surrounding the endogenous ear [13,21,22] to widespread ectopic otic induction at the expense of other sensory organs [23-25]. Infection of chick embryonic hindbrain and surface ectoderm with an *Fgf3*-expression virus vector at early somite stages results in an enlarged otocyst and ectopic otic vesicles just anterior and posterior to the normal otic vesicle [21]. Furthermore, implantation of beads coated with FGF8 or FGF2 close to the developing placode in the chick embryo at early segmentation stages produces a similar result [22]. An enlarged otic placode and enlarged otic vesicle have also been reported in zebrafish after overexpression of Fgf8 by mRNA injections at the 2-cell stage and after implantation of FGF8-coated beads at shield stage [13]. In contrast, injection of 8-cell stage zebrafish embryos with plasmid DNA containing *fgf3 *or *fgf8 *under the control of a constitutive promoter leads to early variegated misexpression of Fgf3 or Fgf8 and ectopic otic induction all around the anterior neural plate border at the expense of other sensory organs [23,24]. Similar results were obtained in *Medaka *after misexpression of Fgf8 under the control of an artificial heat shock promoter at midgastrula stages [25]. Treatment of zebrafish embryos with retinoic acid greatly expands the hindbrain expression domains of *fgf3 *and *fgf8 *and leads to Fgf-dependent formation of ectopic, supernumerary otic vesicles abutting the anterior neural plate [11].

Transplantation experiments suggest that competence of naive ectoderm to form an otic placode is initially widespread but becomes restricted to a region adjacent to the hindbrain by late gastrula or early neurula stages (reviewed in [26,27]). We previously proposed that Foxi1 and Dlx3b may provide the molecular basis for this competence to form an ear [28]. Expression of the homeobox gene, *dlx3b*, is initiated at the beginning of gastrulation on the future ventral side of the embryo and together with *dlx4b*, is restricted in late gastrula stage embryos to a stripe corresponding to cells of the future neural plate border. Expression of both genes is subsequently further restricted to cells of the future olfactory and otic placodes by the beginning of somitogenesis [29-32]. Knockdown of *dlx3b *and *dlx4b *together causes severe loss of otic tissue even in the presence of functional Fgf signaling, consistent with them playing a role to specify the competence of cells to form the ear [33,34]. Expression of the forkhead winged helix transcription factor, *foxi1*, is initiated prior to gastrulation in the future ventral half of the embryo and is progressively restricted to bilateral regions including the preotic domain at late gastrula stages [35-37]. Disruption of *foxi1 *leads to severe defects in otic placode formation and highly variable ear phenotypes [36,37] suggesting that *foxi1 *may also influence otic competence. Loss of both Dlx3b and Foxi1 together ablates all indications of otic induction even in the presence of a fully functional Fgf signaling pathway [24,28]. BMP signaling may regulate early expression of both *dlx3b *and *foxi1 *because *dlx3b *is not expressed in *bmp2b *mutant embryos and overexpression of the Bmp-binding antagonist *noggin *by mRNA injection completely abolishes *foxi1 *expression [38,39]. In addition to BMP, Fgf signaling has also been implicated as a regulator of *foxi1*, although the published results are inconsistent [23,39,40]; Phillips et al. [23] report that localized misexpression of *fgf3 *or *fgf8 *can induce high-level expression of *foxi1 *in the anterior head region, whereas Fürthauer et al. [39] and Kudoh et al. [40] show that Fgf signaling represses *foxi1 *expression.

Here we show that Fgf signaling has different consequences for otic induction depending upon the developmental stage. Due to its repression of non-neural ventral fate before and during gastrulation, Fgf activity early in development leads to ablation or reduction of otic tissue which is foreshadowed by the loss or reduction of *dlx3b *and *foxi1*, respectively. We demonstrate that neither *dlx3b *nor *foxi1 *is activated by Fgf signaling, but rather BMP signaling regulates *dlx3b *indirectly and *foxi1 *directly. Later at the end of gastrulation, however, activation of the Fgf signaling pathway leads to Dlx3b and Foxi1 dependent formation of ectopic otic tissue. Our results support the hypothesis that Foxi1 and Dlx3b provide competence for cells to respond to Fgf signaling and form an otic placode.

## Results

### Misexpression of *fgf8 *induces ectopic otic tissue within the normal otic field

It was recently shown that Fgf signaling is not only necessary but also sufficient for otic induction. Several studies showed that gain of Fgf function results in expanded or ectopic otic induction, however, the location and extent of this effect was inconsistent among the studies [13,21-25]. Differences in the time during development that Fgf signaling was increased in these various studies might explain these inconsistencies.

To control the time of Fgf signaling precisely, we generated a stable transgenic line that allows us to express *fgf8 *uniformly under the control of the zebrafish temperature-inducible *hsp70 *promoter. We tested the reliability of the line by monitoring *fgf8 *expression in transgenic animals before and after heat shock at the end of gastrulation and at midsegmentation stages. Without heat shock, all embryos from a cross between heterozygous *hsp:fgf8 *and wild-type fish show the endogenous pattern of *fgf8 *expression. Following a 30 minute heat shock at either developmental stage, we observe strong and ubiquitous expression of *fgf8 *in 50% of the progeny. Expression levels of *fgf8 *under these conditions are very high and, thus, mask endogenous *fgf8 *expression (additional file 1). Ectopic *fgf8 *mRNA is gradually lost within approximately 180 minutes of its onset, indicating that heat shock leads to strong overexpression of *fgf8 *for a relatively short period of time (additional file 1).

**Figure 1:**
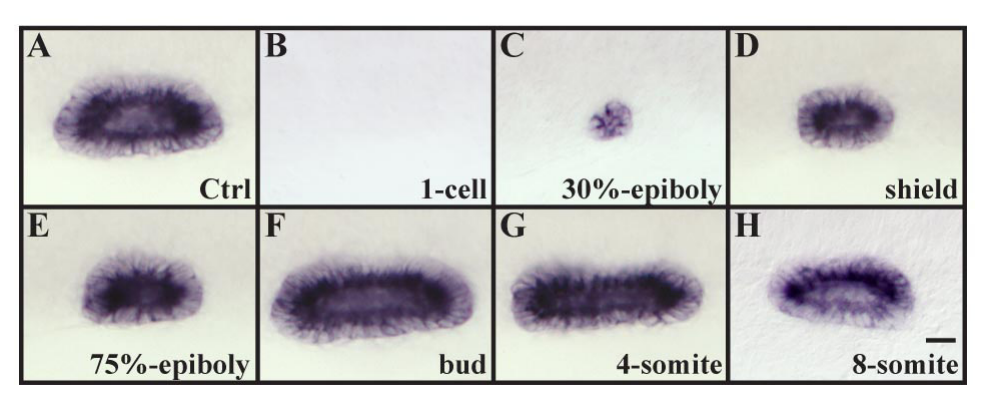
Otic vesicle size is affected by the overexpression of *fgf8 *depending on the developmental stage. (A, B) Overexpression of *fgf8 *by mRNA injections at the 1-cell stage completely ablates all indications of otic fate (27/40 embryos, B) in comparison to wild-type vesicles (A). (C-E) Misexpression of *fgf8 *at 30% epiboly (28/28 embryos, C), shield stage (27/28 embryos, D) and 75% epiboly (29/29 embryos, E) results in smaller otic vesicles. (F-H) Otic vesicles are increased in size if *fgf8 *is misexpressed at the tailbud (26/29 embryos, F) or the 4-somite stage (24/28 embryos, G) whereas at the 8-somite stage no change in vesicle size is observed (26/26 embryos, H). Lateral views of otic vesicles highlighted with *starmaker *at 24h with anterior to the left and dorsal towards the top. Scale bar: 30 μm.

Using this line and *fgf8 *mRNA injections, we subsequently examined the consequences of *fgf8 *misexpression at various times during development as indicated by, *starmaker *(*stm*) that is expressed throughout the otic vesicle epithelium from early vesicle stages. Overexpression of *fgf8 *by mRNA injection at the 1-cell to 2-cell stage leads to severe dorsalization and expansion of the neural plate at the expense of epidermal and preplacodal ectoderm; hence, embryos display severe reduction (32%) or complete loss (68%) of otic tissue (Fig. 1A,B and data not shown). Misexpression of *fgf8 *upon heat shock from late blastula through gastrula stages also causes dorsalization, and embryos show a reduction in the size of the otic vesicle (Fig. 1C,D,E). The size of the otic vesicle that forms is correlated with the time at which *fgf8 *is overexpressed. A very small vesicle forms after *fgf8 *misexpression before gastrulation, whereas later misexpression of *fgf8 *leads to larger otic vesicles. Larger than normal otic vesicles are produced after overexpression of *fgf8 *at the end of gastrulation or during early segmentation stages (Fig. 1F,G), and misexpression at the later time also frequently resulted in duplicated otic vesicles (data not shown). Misexpression of *fgf8 *at even later stages, such as the 8-somite stage, either has no effect on otic vesicle size or produces slightly smaller otic vesicles, presumably due to patterning defects (Fig. 1H). Although *fgf8 *misexpression during gastrulation appears to increase the number of cells fated for otic development, as evidenced by the increase in otic vesicle size, in no case did we observe signs of ectopic otic tissue outside of the normal ear field, that is, outside the field of cells that normally express the putative otic competence factors, *dlx3b *and *foxi1*. We also did not observe formation of ectopic otic vesicles at the expense of other sensory organs. Our results suggest that the ability of ubiquitously expressed Fgf8 to act as an ear inducing factor is restricted to times between the end of gastrulation and early segmentation and to the region where the ear normally forms.

### Misexpression of *fgf8 *affects the expression of *foxi1 *and *dlx3b*

**Figure 2:**
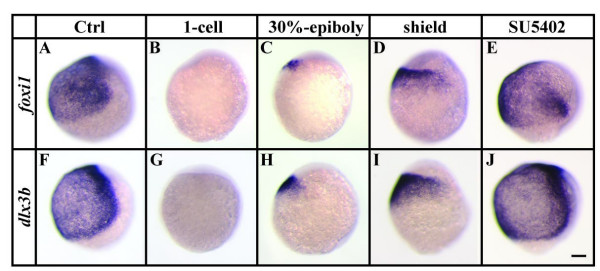
Ectopic Fgf-signaling represses *foxi1 *and *dlx3b *before and during gastrulation. (A, B, F, G) Expression of *foxi1 *and *dlx3b *is absent after overexpression of *fgf8 *by mRNA injections at the 1-cell stage in comparison to wild-type embryos. (C, D, H, I) Misexpression of *fgf8 *at 30% epiboly or shield stages results in smaller *foxi1 *and *dlx3b *expression domains. (E, J) Loss of Fgf-signaling after pharmacological inhibition with SU5402 from late blastula stages until the end of gastrulation has no effect on the ventral expression of *foxi1 *and *dlx3b*. Lateral views at the end of gastrulation with dorsal to the right and anterior towards the top. Scale bar: 100 μm.

We and others recently showed that loss of both Foxi1 and Dlx3b together ablates all indications of otic induction even in the presence of a fully functional Fgf signaling pathway [24,28]. Because both transcription factors are already expressed by gastrulation stages, we hypothesized that otic vesicle loss or reduction after *fgf8 *overexpression might be due to compromised expression of *foxi1 *and/or *dlx3b*. To test this hypothesis, we induced misexpression of *fgf8 *at various times and then fixed embryos at late gastrulation stages when Fgf-dependent otic induction begins. Overexpression of *fgf8 *by mRNA injection at the 1-cell stage, which prevents otic vesicle formation, frequently (90%) leads to embryos showing a complete loss of *foxi1 *and *dlx3b *(Fig. 2A,B,F,G). Misexpression of *fgf8 *at late blastula or early gastrulation stages, which results in smaller otic vesicles at later stages, causes reduced expression of both *foxi1 *and *dlx3b*, showing again a strong correlation between the onset of *fgf8 *overexpression and the size of the *foxi1 *and *dlx3b *expression domains (Fig. 2C,D,F,H,I).

The effect of Fgf8 on Foxi1 and Dlx3b expression is probably due to its early role in dorsoventral patterning. It was recently shown that the balance between Fgf and Bmp signaling regulates dorsoventral patterning of the zebrafish embryo into prospective neural and epidermal domains [39-41]. Furthermore, recent results suggest that Fgf induced activation of MAP kinase is able to interfere with Bmp signaling by phosphorylation of specific residues in the linker region of Smad1, leading to Smad1 inactivation [42,43]. We find that after overexpression of *fgf8*, the reduced *foxi1 *and *dlx3b *expression domains still overlap *p63*, an epidermal marker, and are complementary to *sox3*, a neural marker (data not shown). This indicates that ectopic activation of Fgf signaling at late blastula or early gastrulation stages is still able to activate neural fate at the expense of epidermal fate, hence reducing the size of the *foxi1 *and *dlx3b *expression domains. Additionally, *foxi1 *or *dlx3b *are not expressed ectopically as can happen after localized Fgf misexpression [23].

To provide further insight into the role of Fgf8 signaling in Foxi1 and Dlx3b patterning, we treated wild-type embryos from late blastula stages until the end of gastrulation with SU5402, a specific inhibitor of the tyrosine kinase activity of all Fgf receptors [44]. We find that SU5402, at a concentration of 40 μM, blocks expression of the Fgf-dependent gene, *sprouty4*, (data not shown) indicating that inhibition is complete. Nevertheless, expression of both *foxi1 *and *dlx3b *is unaffected by the inhibitor (Fig. 2E,J). These results show that Fgf signaling is able to repress *foxi1 *and *dlx3b *expression but is not required for their initial activation.

### Early Bmp signaling activates *foxi1 *directly and *dlx3b *indirectly

**Figure 3:**
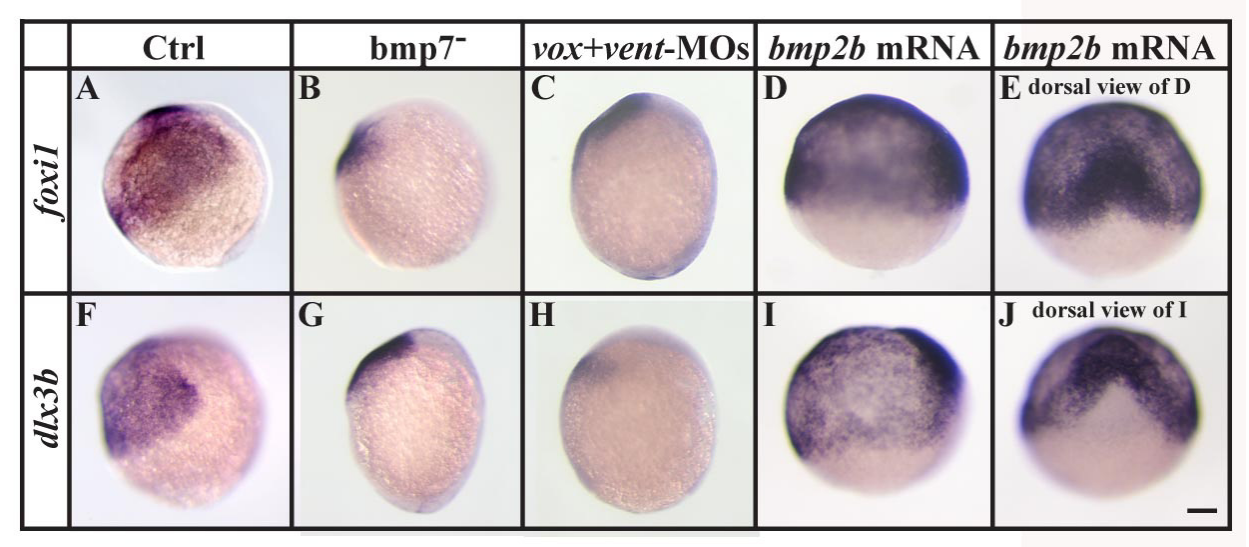
Bmp-signaling is required for *foxi1 *and *dlx3b *expression during gastrulation. (A-C, F-H) Expression of *foxi1 *and *dlx3b *is strongly reduced in *bmp7 *mutants or embryos injected with Vox and Vent morpholinos (MOs) in comparison to wild-type embryos. (D, E, I, J) Overexpression of *bmp2b *by mRNA injections at the 1-cell stage leads to an expansion of both *foxi1 *and *dlx3b *expression domains at the expense of anterior neural plate. (A-D, F-I) Lateral views at the end of gastrulation with dorsal to the right and anterior upward; (E, J) dorsal views of embryos shown in D and I with anterior towards the top. Scale bar: 100 μm

Our *fgf8 *overexpression studies showed that *foxi1 *and *dlx3b *are negatively regulated in dorsal ectoderm by Fgf signaling, raising the possibility that Bmp signaling might activate these genes in the ventral ectoderm. In support of this idea, we find that neither *foxi1 *nor *dlx3b *is expressed in *bmp2b *mutants (data not shown) and their expression is strongly reduced in *bmp7 *mutants (Fig. 3A,B,F,G). We also observe the same phenotype after knockdown of the ventralizing transcriptional repressors, Vox and Vent, by antisense morpholino oligonucleotides (Fig. 3C,H). Furthermore, overexpression of Bmp2b by mRNA injection expands expression of *foxi1 *and *dlx3b *at the expense of anterior neural plate (Fig. 3D,E,I,J).

**Figure 4:**
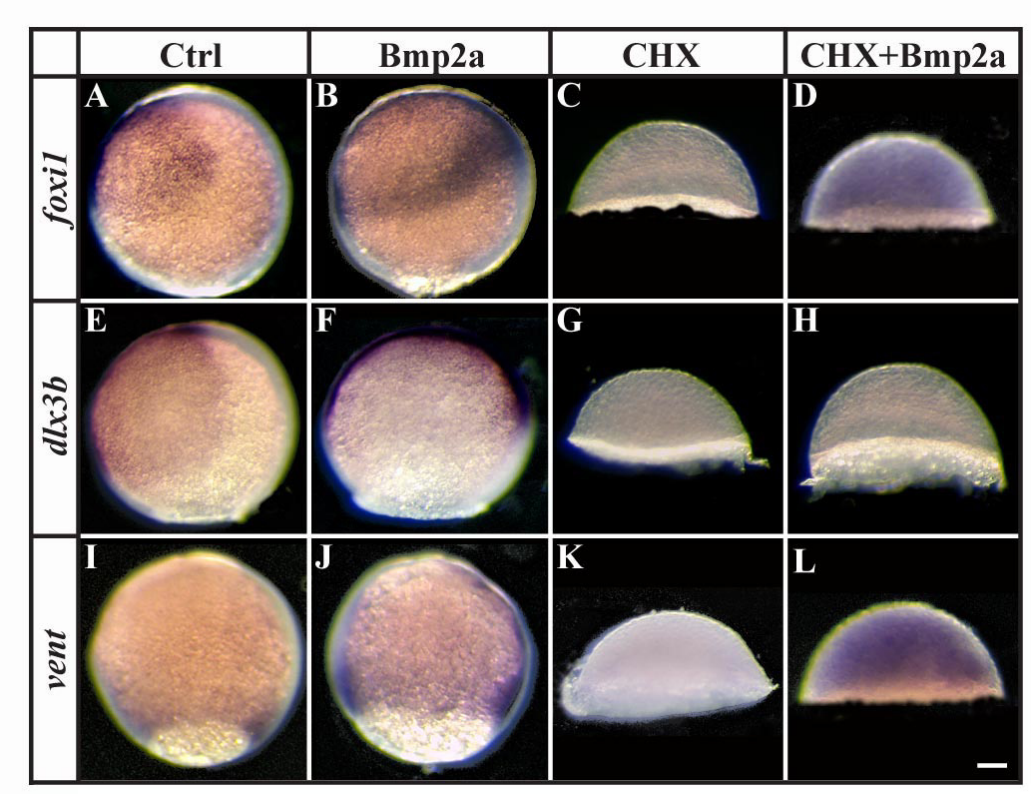
*foxi1 *and *vent *but not *dlx3b *are direct targets of Bmp-signaling during gastrulation. (A, B, E, F, I, J) Expression of *foxi1*, *dlx3b *and *vent *is expanded after Bmp2a protein injection at late blastula stages in comparison to wild-type embryos. (C, D, G, H, K, L) Pharmacological inhibition of protein synthesis with cycloheximide (CHX) at late blastula stages blocks all indications of *foxi1*, *dlx3b *and *vent *expression (C, G, K), whereas *foxi1 *and *vent *but not *dlx3b *expression is restored in CHX-treated embryos after Bmp2a protein injection (D, H, L). Lateral views at the end of gastrulation with dorsal to the right and anterior towards the top (note that CHX treatment blocks epiboly movements). Scale bar: 90 μm.

To learn whether *foxi1 *and *dlx3b *are direct transcriptional targets of Bmp, we injected embryos at late blastula stages (sphere) with Bmp2a protein and raised them in cycloheximide (CHX), a protein synthesis inhibitor. As a positive control, we examined expression of *vent*, a direct target of Bmp signaling [45,46]. As expected, *vent *is ectopically expressed after Bmp2a protein injection (Fig. 4I,J). Control or BSA-injected embryos treated with CHX have arrested gastrulation movements and do not exhibit *vent *expression (Fig. 4K). In CHX-treated embryos injected with Bmp2a protein, however, gastrulation movements are still blocked but *vent *expression is restored indicating that Bmp2a protein is sufficient for *vent *expression and *de novo *protein synthesis is not required (Fig. 4L). Both *foxi1 *and *dlx3b *behave similarly to *vent *with ectopic or missing expression after Bmp2a protein injection or CHX treatment, respectively (Fig. 4A–C, E–G). However, after Bmp2a protein injection and CHX treatment, *foxi1 *expression is restored, like *vent*, whereas *dlx3b *expression is not (Fig. 4D,H). Thus, our results suggest that Bmp signaling activates both *foxi1 *and *dlx3b *expression. Furthermore, as early as sphere stage, *foxi1*, but not *dlx3b*, acts as a direct transcriptional target of the Bmp signaling pathway.

### Misexpression of *fgf8 *at late gastrulation stages leads to expanded induction of otic markers within the preotic field

**Figure 5:**
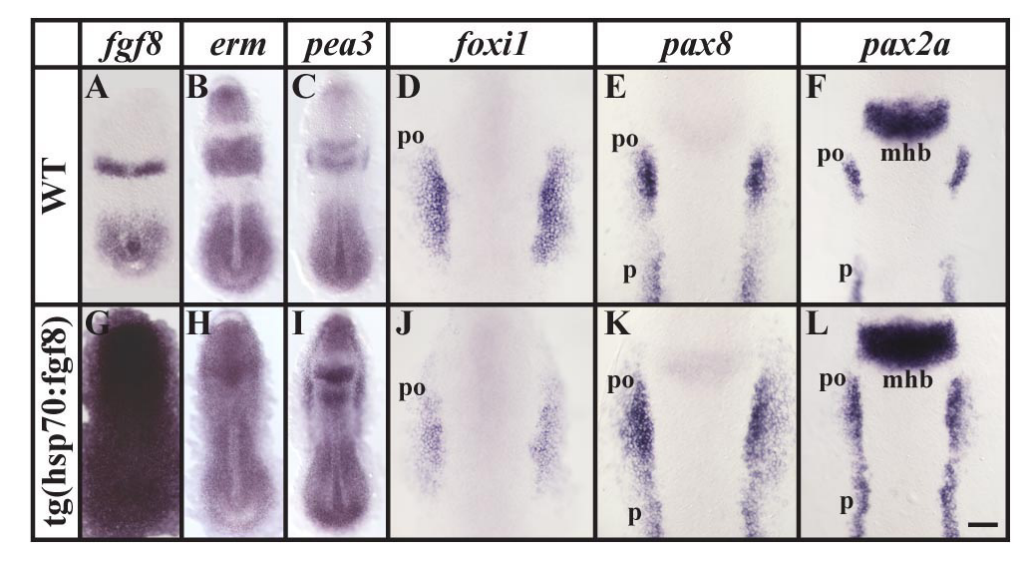
Ubiquitous *fgf8 *expression at late gastrulation stages leads to ectopic otic induction within the preotic field. (A, G) Ectopic *fgf8 *expression can be detected throughout the embryo after a 30 minute heat shock in transgenic *hsp:fgf8 *embryos in comparison to non-transgenic embryos. (B, C, H, I) Within 2 hours, expression of the two Fgf reporter genes, *erm *and *pea3*, is upregulated in cells of the transgenic embryos whereas non-transgenic siblings are unaffected. (D-F, J-L) After misexpression of *fgf8 *at late gastrulation stages, expression of *foxi1 *is reduced, whereas the preotic expression domains of *pax8 *and *pax2a *are enlarged. Dorsal views of 2–5-somite stage embryos with anterior towards the top. mhb, midbrain-hindbrain border; po, preotic region; p, pronephros. Scale bar: 100 μm for A-C, G-I; 40 μm for D-F, J-L.

To understand how overexpression of *fgf8 *at the end of gastrulation or early segmentation stages leads to the formation of much larger otic vesicles, we examined early markers of otic induction. First, to learn whether all cells receive the ectopic Fgf signal, we examined expression of two Fgf reporter genes, *erm *and *pea3*, members of the ETS family of transcription factors [47,48]. We find that within 90 minutes after a 30 minute heat shock at the end of gastrulation or during early segmentation stages, essentially all cells of the transgenic embryo respond to the ubiquitous Fgf signal whereas non-transgenic siblings are unaffected (Fig. 5B,C,H,H and data not shown). Expression of *foxi1 *that is restricted to two bilateral domains at this stage is again reduced, whereas *dlx3b *expression surrounding the anterior neural plate is unchanged (Fig. 5D,J and data not shown). This indicates that *foxi1 *but not *dlx3b *is presumably still under the control of Bmp signaling that is attenuated by the anti-Bmp activity of elevated Fgf8 in heat shocked transgenic embryos. In contrast to the reduced *foxi1 *expression, we find that expression of the early preotic marker, *pax8*, a paired box homeodomain gene, is expanded but only within the preotic region (Fig. 5E,K). In wild-type embryos at the one-somite stage, double labeling with both *foxi1 *and *pax8 *shows that preotic, Fgf-dependent *pax8 *is expressed only in part of the *foxi1 *expression domain [11-13,36]. After exposure of the entire *foxi1*-positive domain to *fgf8 *in transgenic embryos, Foxi1 protein, present prior to the heat shock, presumably persists and supports the observed expanded *pax8 *expression. Because preotic *pax8 *expression depends completely on Foxi1, and *foxi1 *expression is restricted to two bilateral domains but is not expressed in other regions of the preplacodal domain at the end of gastrulation where we observe expanded *pax8*, we also examined expression of *pax2a*, another early marker of otic fate. *pax2a *expression depends partially on *foxi1 *and partially on *dlx3b *that is expressed in a stripe corresponding to cells of the future neural plate border, which presumably represents the entire preplacodal domain [34]. As with *pax8*, we detect expanded *pax2a *expression but again only in the preotic region of heat shocked, transgenic embryos (Fig. 5F,L). Examination of *sox9a *expression [34], a third early otic marker, yields identical results with upregulation of *sox9a *only in the preotic region but not in other regions of the preplacodal domain of transgenic embryos (additional file 2C, F). Together, these results show that cells only in the preotic region are competent to embark upon the path towards otic fate even if Fgf signaling is strongly activated throughout the entire embryo.

### Ectopic otic cells undergo normal ear development

**Figure 6:**
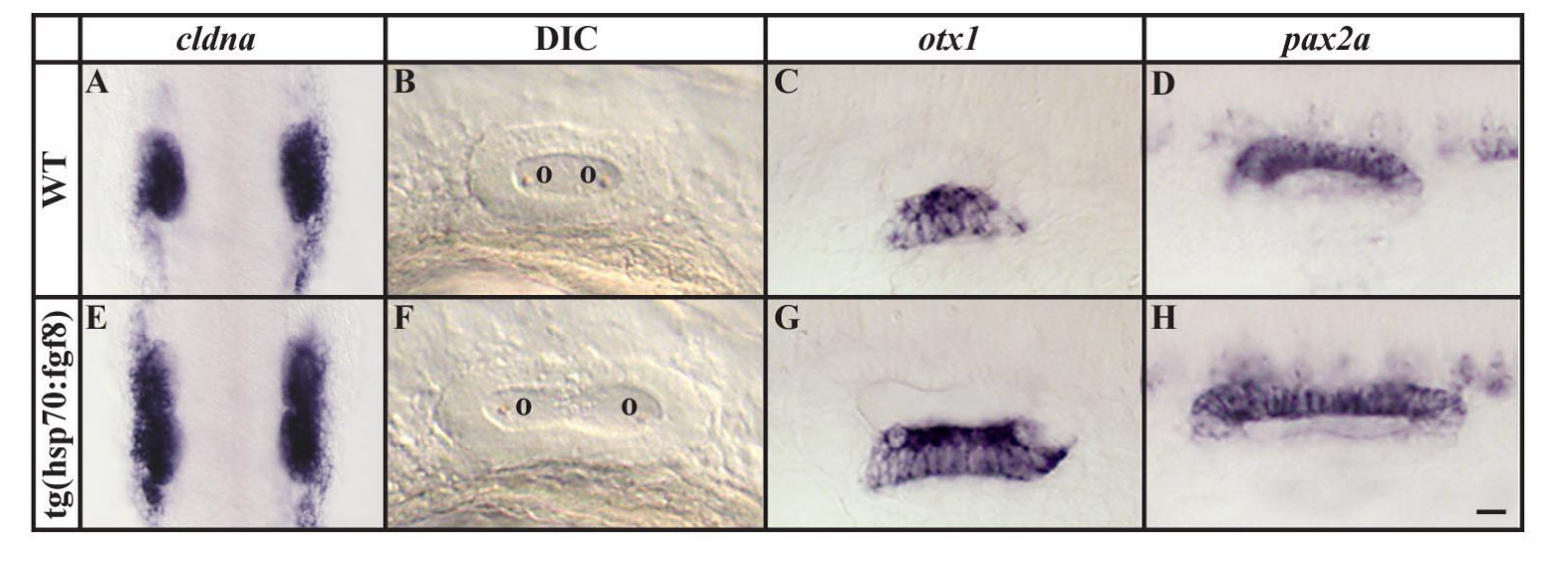
Ectopic otic induction after *fgf8 *expression at late gastrulation stages leads to formation of larger but correctly patterned placodes and vesicles. (A, E) Otic placodes labeled by *cldna *expression are increased in size in transgenic *hsp:fgf8 *embryos in comparison to non-transgenic embryos following a heat shock at late gastrulation stages. (B-D, F-H) The enlarged otic vesicles of transgenic embryos show no apparent patterning defects in comparison to non-transgenic siblings assessed by morphology and marker gene expression, including *otx1 *and *pax2a*. (A, E) Dorsal views of 12-somite stage embryos with anterior towards the top; (B-D, F-H) lateral views of otic vesicles at 24h with anterior to the left and dorsal towards the top. o, otolith. Scale bar: 50 μm for A, E; 30 μm for B-D, F-H.

Because the expression domains of *pax8 *and *pax2a *are expanded in the preotic region in the transgenic embryos, we examined whether these cells undergo normal development. We find that many more cells contribute to the otic placode in heat shocked, transgenic animals than in non-transgenic siblings (Fig. 6A,E). Nevertheless, the otic vesicle subsequently forms with no overt morphological differences other than its increased size. Both transgenic and non-transgenic embryos form two otolith seedlings and no size difference can be observed after three days of development (Fig. 6B,F and additional file 3). Moreover, patterning of the otic vesicle developed normally as indicated by *fgf8*, *hmx3 *and *follistatin *that demarcate anteroposterior domains, by *dlx3b*, *otx1 *and *wnt4 *in dorsoventral domains and by *pax2a *that highlights mediolateral domains at 24 h (Fig. 6C,D,G,H and data not shown).

### Foxi1 and Dlx3b both participate in expanded otic induction

**Figure 7:**
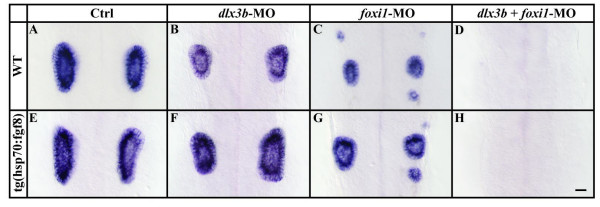
Ectopic otic induction after *fgf8 *expression at late gastrulation stages requires both, Foxi1 and Dlx3b. (A-D) Inactivation of Dlx3b or Foxi1 in wild-type embryos by morpholino injection (MO) leads to a reduction of ear size in comparison to wild-type embryos, and combined loss of Dlx3b and Foxi1 results in loss of all indications of otic specification. (E-H) Ear size reduction by depletion of Dlx3b or Foxi1 but not combined loss of Dlx3b and Foxi1, can be partially rescued in transgenic *hsp:fgf8 *embryos heat shocked at late gastrulation stages. Lateral views of otic vesicles hybridized with *starmaker *at 24h with anterior to the left and dorsal towards the top. Scale bar: 40 μm.

Because ectopic expression of *pax8 *and *pax2a*, in response to overexpression of *fgf8*, is confined to the preotic region and overlaps with the expression domain of *foxi1 *but not *dlx3b*, it is possible that ectopic induction depends exclusively on Foxi1. We recently showed that depletion of Foxi1 or Dlx3b alone results in formation of a reduced otic vesicle and that removal of both factors leads to complete failure of otic specification [28]. We thus examined the response of embryos to *fgf8 *overexpression in the absence of either Dlx3b or Foxi1. We find that overexpression of *fgf8 *at the end of gastrulation rescues the small otic vesicle generated by compromised Dlx3b function (Fig. 7A,B,E,F). Surprisingly however, we also detected partial rescue by misexpression of *fgf8 *in embryos depleted of Foxi1 (Fig. 7C,G). Consistent with our previous results [28], we are unable to detect any sign of otic specification after loss of both factors, Foxi1 and Dlx3b, even if Fgf signaling is significantly increased (Fig. 7D,H). Taken together, our results show that either Foxi1 or Dlx3b is required for cells to be competent to respond to Fgf8 signaling and subsequently form the ear. Loss of either Foxi1 or Dlx3b can be partially compensated by an increase in Fgf signaling, but removal of both leads to complete loss of otic fate.

### Ectopic *foxi1 *provides competence for ectopic Fgf-dependent otic induction

**Figure 8:**
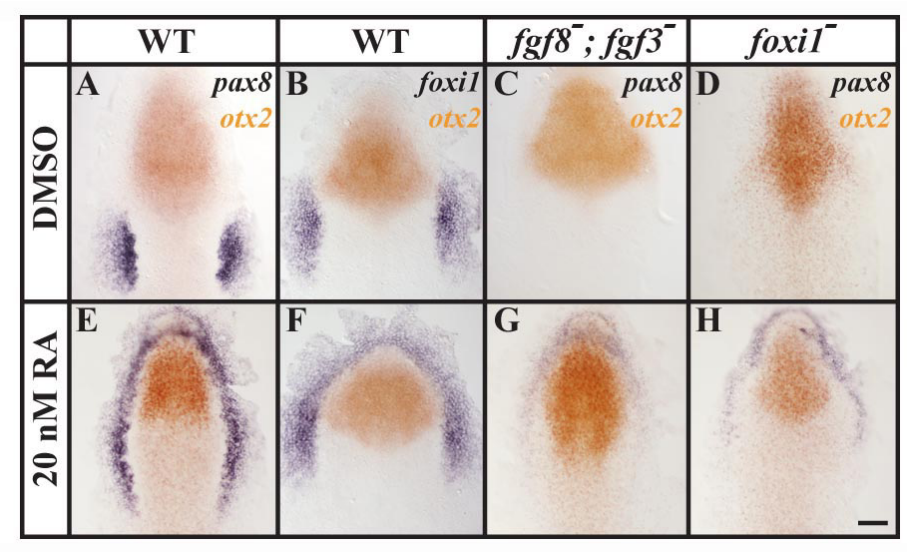
Ectopic *foxi1 *expression after treatment with retinoic acid (RA) results in ectopic Fgf-dependent otic induction. (A, B, E, F) In comparison to wild-type control embryos treated with DMSO, embryos treated with 20nM RA show ectopic *pax8 *and *foxi1 *expression surrounding the anterior neural plate border without affecting the neural expression of *otx2*. (C, G) In *fgf3*, *fgf8 *double mutants, *pax8 *is completely abolished in the control embryos, whereas RA-treated double mutant embryos show weak anterior expression of *pax8*. (D, H) In *foxi1 *mutants treated with DMSO, *pax8 *expression can not be detected in the preotic region; but in *foxi1 *mutants treated with RA, residual anterior *pax8 *expression is present. Dorsal views of 1–3-somite stage embryos with anterior towards the top. Scale bar: 40 μm.

To examine whether Foxi1 expression is sufficient for cells to be competent to adopt an otic fate, we used several different approaches. Misexpression of *foxi1 *by mRNA or plasmid injections at the 1- to 2-cell stage in *hsp:fgf8 *embryos or transplantation of *foxi1 *positive cells into *hsp:fgf8 *host embryos followed by heat shock at late gastrulation stages were inconclusive (data not shown). Instead, we used retinoic acid (RA) treatment to expand the *foxi1 *expression domain. Application of high doses of RA (1 μM) results in expansion of the hindbrain expression domains of *fgf3 *and *fgf8 *throughout the anterior neural plate and massive, Fgf-dependent expansion of the *pax8 *expression domain [11]. We find that a much lower concentration of RA (20 nM) is sufficient to expand *pax8 *expression around the anterior border of the neural plate (Fig. 8A,E). Embryos treated with 20nM RA show no change in *otx2*, *fgf3*, *fgf8 *or *sox9a *expression, indicating that this RA concentration does not posteriorize the anterior neural plate or expand posterior expression domains (Fig. 8 and additional file 2). Consistent with this, RA treated wild-type embryos show no loss of anterior structures but develop enlarged otic vesicles at 1 and 2 days of development (unpublished results). At the end of gastrulation, *dlx3b *expression in RA treated embryos is indistinguishable from untreated embryos (data not shown), whereas the *foxi1 *expression domain is greatly expanded. We find that *foxi1 *is no longer limited to the two bilateral domains but instead expands into the entire preplacodal domain similar to the *dlx3b *stripe surrounding the anterior neural plate (Fig. 8B,F,). In wild-type embryos, *pax8 *expression depends upon Fgf signaling [11-13], as confirmed by use of *fgf3*, *fgf8 *double mutants (Fig. 8C). In *fgf3*, *fgf8 *double mutants treated with RA, *pax8 *expression is greatly reduced, although we find some residual anterior expression (Fig. 8G), suggesting that a remaining Fgf (perhaps Fgf24, Fgf17a or Ff17b; [49-51]) may provide inductive signaling for *pax8 *expression in this region. In *foxi1 *mutants preotic *pax8 *expression is completely abolished, whereas in RA treated *foxi1 *mutants *pax8 *expression is largely gone, although surprisingly we find some residual expression at the anterior end (Fig. 8D,H). Depletion of Dlx3b and Dlx4b by morpholino injections in RA-treated *foxi1 *mutants did not further reduce *pax8 *expression and we also could not detect any ectopic expression of the three known members of the zebrafish Foxi class genes, *foxi2*, *foxi3a *and *foxi3b *(data not shown). The persistence of some residual *pax8 *expression in RA treated *foxi1 *mutant embryos indicates that additional genes might be involved in otic induction in the presence of RA. However taken together, our results show that most of the ectopic otic induction in RA treated embryos is due to the presence of ectopic Foxi1.

## Discussion

### The competence of preotic cells to respond to Fgf-signaling is acquired at the end of gastrulation

Numerous loss of function and gain of function studies have led to the conclusion that Fgf signaling is necessary and sufficient for induction of the otic placode, although not all cells are competent to respond to the inductive signal. Our results add several pieces of evidence in support of our hypothesis [28] that competence results from expression of Foxi1 or Dlx3b transcription factors: 1) Cells become competent shortly after they start expressing the factors (Fig. 1). 2) Expanding the domain of Fgf signaling extends otic fate to cells that express the factors but normally see only low levels of signal (Fig. 6), but not to cells that never express the factors (Fig. 5). 3) Cells that ectopically express Foxi1 acquire competence (Fig. 8), whereas 4) cells that lose both factors lose competence (Figs. 1, 2, 3), even when Fgf signaling is elevated (Fig. 7).

**Figure 9:**
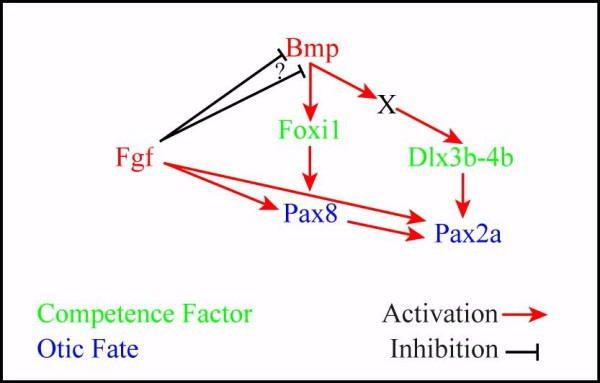
Model summarizing the early events upstream of otic induction. During gastrulation, Fgf signaling antagonizes Bmp signaling that activates the competence factors Foxi1 and Dlx3b-4b (green). Foxi1 is activated by BMP signaling in a direct manner whereas Dlx3b-4b requires an additional factor X. It is unknown whether Fgf signaling inhibits Bmp signaling at a transcriptional or translational level (?). The proper balance of Bmp and Fgf signaling during gastrulation promotes the positioning and size of the preotic region leading to the induction of otic fate as indicated by Pax8 and Pax2a expression (blue).

Our results further show that there is only a relatively short time window for otic induction, between the end of gastrulation and early segmentation stages. Misexpression of *fgf8 *only within this period leads to formation of enlarged otocysts whereas overexpression earlier or later results in smaller or unchanged otic vesicles (Fig. 1). Smaller otic vesicles after misexpression of *fgf8 *up to midgastrulation stages is presumably due to the effects of Fgf signaling on dorsoventral patterning that subsequently affects expression of the Foxi1 and Dlx3b expression domains. Fgf and Bmp signals antagonistically control dorsoventral patterning of the embryo during gastrulation and are necessary to establish prospective neural and epidermal domains [39-41]. Consistent with this interpretation, we see that ectopic Fgf signaling leads to an expanded neural domain and a reduced epidermal domain (Fig. 2). The reduction in size of the epidermal domain results in smaller Foxi1 and Dlx3b expression domains, and, hence, reduced numbers of cells that are competent to form the ear (Fig. 9).

### Foxi1 and Fgf signaling act together to regulate *pax8 *expression

Transplantation experiments suggest that competence of naive ectoderm to form an otic placode is initially widespread during early gastrulation stages but becomes subsequently restricted to a relatively small area abutting the hindbrain at early segmentation stages [26,27]. The expression patterns of the *foxi1 *and *dlx3b *genes show similar progressive restrictions during early development consistent with the proposed roles of Foxi1 and Dlx3b proteins as the molecular regulators of otic competence [28]. Expression of both *foxi1 *and *dlx3b *is initiated prior to or at the beginning of gastrulation throughout the future ventral side of the embryo. Subsequently *foxi1 *expression is restricted to bilateral domains at the lateral edges of the hindbrain while *dlx3b *is restricted to a stripe of cells corresponding to the future neural plate border, including the preotic domain [29-32,36,37,52]. Depletion of *foxi1 *or *dlx3b *leads to loss of otic tissue and the combined loss of both, Dlx3b and Foxi1, ablates all indications of otic induction even in the presence of a functional Fgf signaling pathway [24,28,33,34]. The changes in *foxi1 *and *dlx3b *expression after misexpression of *fgf8 *up to midgastrulation stages (Fig. 2) foreshadows the loss of otic tissue, providing further evidence that these genes are required within the preotic region for otic induction. We never observe ectopic expression of *foxi1 *or *dlx3b *after misexpression of *fgf8 *and further demonstrate that *foxi1 *and *dlx3b *are positively regulated by Bmp signaling but not Fgf signaling (Fig. 2, 3).

These results contrast with a recent study that reported ectopic expression of *foxi1 *after Fgf misexpression [23]. The conflicting results are presumably due to differences in the methods used to obtain ectopic Fgf signaling. Phillips et al. [23] injected 8-cell stage embryos with plasmid DNA encoding Fgf3 or Fgf8 under the control of a constitutive promoter. This leads to early variegated misexpression [53] of Fgf3 or Fgf8. With this technique, embryos exhibited some moderate dorsalization, but by co-labeling for neural markers and Fgf expression, the authors ensured that the analyzed embryos had only scattered patches of Fgf expressing cells and no overt signs of dorsalization. However, even with no overt signs of dorsalization, patchy activation of Fgf signaling during gastrulation creates local dorsalizing centers that subsequently alter gastrulation movements resulting in the formation of a partial secondary axis [54]. Use of our *hsp:fgf8 *transgenic line also changes dorsoventral identity but due to strong, uniform expression of *fgf8 *throughout the entire embryo, all cells are exposed to the same level of Fgf signaling. Consequently, no local dorsalizing center is created and no partial secondary axis is generated. Furthermore, strong but relatively brief activation of the Fgf signaling pathway in the *hsp:fgf8 *transgenic line presumably reveals the immediate, direct consequences of ectopic Fgf signaling, whereas prolonged activation is known to affect dorsal ventral patterning, resulting in secondary effects.

Additional support for our interpretation is provided by the complementary expression patterns of Fgfs and *foxi1*. In zebrafish, *fgf3*, *fgf8*, *fgf17b *and *fgf24 *are all expressed in a dorsoventral gradient in the margin that later forms the germring of the gastrulating embryo, with highest levels on the dorsal side [39,51,54,55]. In contrast, *foxi1 *and *dlx3b *are strongly expressed on the ventral side. Expression of the downstream targets of Fgf signaling, *sprouty2 *and *sprouty4*, is limited to the margin [39], indicating that Fgf signaling normally does not extend significantly into the ventral part of the embryo.

Our findings have profound consequences for the placement of *foxi1 *in the genetic pathway regulating otic development. Expression of *foxi1 *in the preotic region has been considered the earliest marker of otic induction [10,56], but we show that *foxi1 *expression does not depend on Fgf signaling, the inducer of otic fate. Consistent with our previous model [28], we propose instead that Foxi1 (competence) and Fgf signaling (induction) represent two independent pathways that act in concert to activate *pax8*, which therefore is one of the earliest indicator of otic induction (Fig. 9).

### Otic competence within the preplacodal region

Misexpression of *fgf8 *at the end of gastrulation leads to ectopic otic induction in a Foxi1 and Dlx3b dependent manner, but only within the preotic region. Treatment of wild-type embryos with RA, however, extends otic induction outside the normal preotic region to include the entire preplacodal domain that gives rise to all the cranial placodes. We show that most but not all of this ectopic otic induction is due to the misexpression of *foxi1*. Although misexpression of *foxi1 *promotes otic induction, the presence of Foxi1 and Dlx3b in non-neural cells alone is insufficient to initiate otic fate in response to Fgf signaling. During gastrulation, *foxi1 *and *dlx3b *are co-expressed in cells on the ventral side of the embryo, but we never observe *pax8 *expression in these cells even when Fgf is ectopically expressed in this region (data are not shown). However, at these stages the preplacodal domain, which represents the earliest stage of placode induction and which is required for otic induction [5,7], has not yet been established. Strong ventral Bmp activity could be responsible for this limitation; a recent study suggests that attenuation of Bmp signaling is required for formation of the preplacodal region [6].

Our results show that not only *fgf3 *and *fgf8 *can act as otic inducers; we still detect some otic induction in *fgf3*, *fgf8 *double mutants treated with RA (Fig. 8). Candidates for this ectopic otic induction in the absence of *fgf3 *and *fgf8 *include *fgf17a*, *fgf17b *and *fgf24 *that are expressed at the anterior end of the embryo at bud stage [50,51,57].

## Conclusion

Taken together our results reconcile the conflicting reports of ectopic otic induction after misexpression of Fgf. Irrespective of the species, a region abutting the hindbrain is competent to adopt the otic fate by the end of gastrulation and until early segmentation stages. In zebrafish, this region of competence is defined by Foxi1 and Dlx3b whereas in other species, the network upstream of Pax8 is still unknown. In chick, no *Foxi1 *genes have been reported and in mouse *Foxi1 *is expressed only very late during otic development, which might explain why disruption of *Foxi1 *causes only a mild otic phenotype without affecting early patterning or morphogenesis [58,59]. Recent analysis of mouse Foxi class genes has revealed that Foxi2 and Foxi3 are expressed in the preotic region during otic induction [60]; thus these factors may provide otic competence in mouse similar to Foxi1 in zebrafish. At least one example of such a switch in function between orthologous genes in zebrafish and tetrapods has already been described [61] supporting the notion that a genetic network similar to what we have proposed for zebrafish [28] may regulate ear development in all vertebrates.

## Methods

### Animals

Embryos, obtained from the University of Oregon zebrafish facility, were produced using standard procedures [62] and were staged according to standard criteria [63] or by hours post fertilization at 28°C (h). The wild-type line used was AB. The mutant lines, *acerebellar*^*ti282a*^, a strong hypomorphic allele of *fgf8*, *snailhouse*^*ty68a*^, a temperature-sensitive allele of *bmp7*, *hearsay*, a presumptive null allele of *foxi1 *and *lia*^*t*2414^, a hypomorphic allele of *fgf3*, have been described previously [33,64-66] and we refer to the homozygous mutants as *fgf8*, *bmp7*, *foxi1 *and *fgf3*, respectively. *bmp7 *embryos and their siblings were raised at 33°C [65].

### Germline transformation

To generate the *hsp70:fgf8 *construct, DNA fragments containing the zebrafish temperature-inducible *hsp70 *promoter [67] and the *fgf8 *gene [50] were cloned into the CS2+ vector [68]. Preparation of the DNA construct for injection followed previous methods [69]. Injected, putative founder fish (F0) were crossed inter se and their progeny (F1) were screened by PCR using *hsp70 *(5' CCC CGA CGA GGT GTT TAT TCG CTC 3') and *fgf8 *(5' CGG GGA CTG AAT GGT TAC CTG AGC 3') primers with the following PCR conditions: 3 minutes at 94°C; 35X (30 seconds at 94°C, 1 minute at 65°C, 1 minute at 72°C); 8 minutes at 72°C. For heat shock treatment, embryos still in their chorions were incubated in 37–39°C embryo medium in a 1.5 ml tube (20 embryos per tube) for 30 minutes in a heating block.

### In situ hybridization, mRNA synthesis and microinjections

cDNA probes that detect the following genes were used: *dlx3b *[30]; *erm *[48]; *fgf8 *[50]; *foxi *[36]; *otx1 *[70]; *otx2 *[70]; *pax8 *[71]; *pea3 *[48]; *stm *[72]; *vent *[46]; *cldna *[73]; *pax2a *[74]. Probe synthesis and single or double color *in situ *hybridization was performed essentially as previously described [75-77]. We purified the *in vitro *synthesized mRNA and probes using an RNeasy mini column (Qiagen GmbH). *In vitro *mRNA synthesis was performed using an SP6 RNA synthesis kit (Ambion). mRNAs were injected into all blastomeres of embryos at the 1-cell to 2-cell stage. RNA concentrations used for injections were: *fgf8 *(80 pg) and *bmp2b *(50 pg). Exogenous Bmp activity was provided by injection of mature, heterodimeric Bmp-2a (R&D Systems, 111-BM). At sphere stage (4 h), 6–8 μl of 200 ng/μl Bmp-2a (in PBS, 0.1% BSA, with phenol red added) was injected into the center of the blastoderm. More than 20 embryos were examined in each injection experiment.

### Morpholinos (MOs) and pharmacological treatments

The *dlx3b*-MO, *foxi1*-MO *vent*-MO and *vox*-MO have been previously described [33,34,78]. For pharmacological treatments, the following stock solutions were made and stored at -80°C: 1 mM all-trans retinoic acid (RA; Sigma) in DMSO; 40 mM SU5402 (Calbiochem) in DMSO; 100 mg/ml cycloheximide (CHX; AG Scientific Inc., # 1189) in Ethanol. For embryo treatments, dilutions of these chemicals were made in embryo medium as follows: retinoic acid: 20 nM; SU5402: 40 μM; cycloheximide: 10^-4^M. Prior to gastrulation embryos were removed from their chorions and transferred into petri dishes containing the treatment solution, except for SU5402 treatments that were done in smaller volumes in glass vials. For control treatments, sibling embryos were incubated in corresponding dilutions of DMSO or Ethanol. CHX-treated embryos were fixed when control siblings reached 85–100% epiboly. All incubations were conducted in the dark.

## Authors' contributions

SH conceived the study, carried out most of the experiments, analyzed the data and drafted the manuscript. JC contributed design and execution of the Bmp2a protein overexpression and cycloheximide experiments, and helped draft the manuscript. DL contributed to the design of the experiments and helped to draft the manuscript. MW, contributed to design and coordination of the experiments, and helped draft the manuscript. All authors have read and approved the final manuscript.

## Supplementary Material

Additional file 1 Heat shock at the end of gastrulation produces strong and ubiquitous *fgf8 *expression in transgenic animals. (A-C, G-K) Following a 30 minute heat shock, strong and ubiquitous expression of *fgf8 *can be observed in the transgenic embryos (G-K) up to 1.5 hours after heat shock, masking the endogenous *fgf8 *expression domains that are observed in the wild-type embryos (A-C). (D, I) Ectopic *fgf8 *mRNA is gradually lost and the endogenous *fgf8 *expression domains emerge at 2 hours after heat shock. (D, I) At 2.5 hours after heat shock only scattered cells show ectopic fgf8 expression in transgenic embryos. Dorsal views of 2–5-somite stage embryos with anterior towards the top. h, hours after heat shock; mhb, midbrain-hindbrain border; tb, tail bud. Scale bar: 100 μm. Click here for file

Additional file 2 Retinoic acid treatment has little effect on patterning along the anterior-posterior axis. (A, B, D, E) Expression of *fgf3 *or *fgf8 *in embryos treated with 20 nM RA is indistinguishable from control embryos treated with DMSO. (C, F) In RA treated embryos expression of *sox9a *in the preotic region expands to surround the anterior neural plate border in comparison to control embryos. However, *sox9a *expression in the hindbrain is identical in RA and DMSO treated embryos. Dorsal views of 1–5-somite stage embryos with anterior towards the top. fb, forebrain; hb, hindbrain; hp, heart primordium; mhb, midbrain-hindbrain border; r4, rhombomere 4; po, preotic region. Scale bar: 40 μm μm. Click here for file

Additional file 3 The increase in otic tissue, following a heat shock at late gastrulation stages of transgenic *hsp:fgf8 *embryos, is transient. (A, D) At 28 hours post fertilization, otic vesicles in transgenic fish heat shocked at late gastrulation stages are still larger than in non-transgenic siblings. (B, C, E, F) The size difference of otic vesicles in transgenic and non-transgenic embryos is less prominent at 50 hours post fertilization, and indistinguishable by 72 hours post fertilization. Lateral views of live otic vesicles with anterior to the left and dorsal towards the top. o, otolith. Scale bar: 120 μm. Click here for file
