# Supplementary figures and images for: Fgf-dependent otic induction requires competence provided by Foxi1 and Dlx3b
Source: BMC Dev Biol. 2007 Jan 19;7:5. doi: 10.1186/1471-213X-7-5 (PMC1794237; doi:10.1186/1471-213X-7-5)

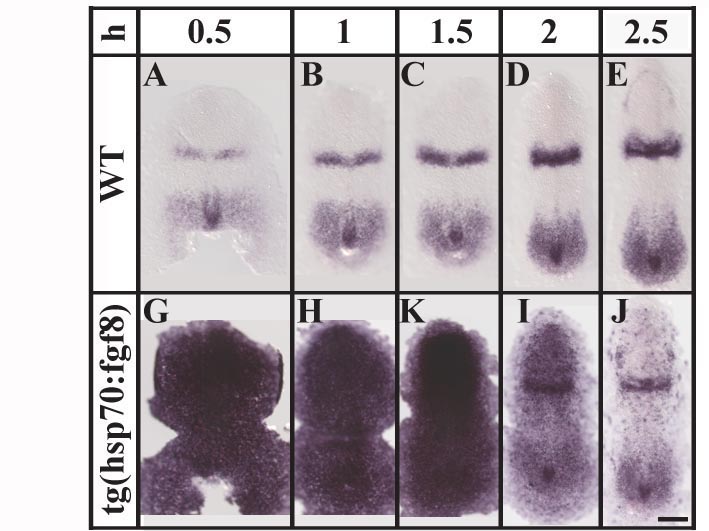

Supplement: Additional file 1 — Heat shock at the end of gastrulation produces strong and ubiquitous fgf8 expression in transgenic animals. (A-C, G-K) Following a 30 minute heat shock, strong and ubiquitous expression of fgf8 can be observed in the transgenic embryos (G-K) up to 1.5 hours after heat shock, masking the endogenous fgf8 expression domains that are observed in the wild-type embryos (A-C). (D, I) Ectopic fgf8 mRNA is gradually lost and the endogenous fgf8 expression domains emerge at 2 hours after heat shock. (D, I) At 2.5 hours after heat shock only scattered cells show ectopic fgf8 expression in transgenic embryos. Dorsal views of 2–5-somite stage embryos with anterior towards the top. h, hours after heat shock; mhb, midbrain-hindbrain border; tb, tail bud. Scale bar: 100 μm. [file 1471-213X-7-5-S1.jpeg]

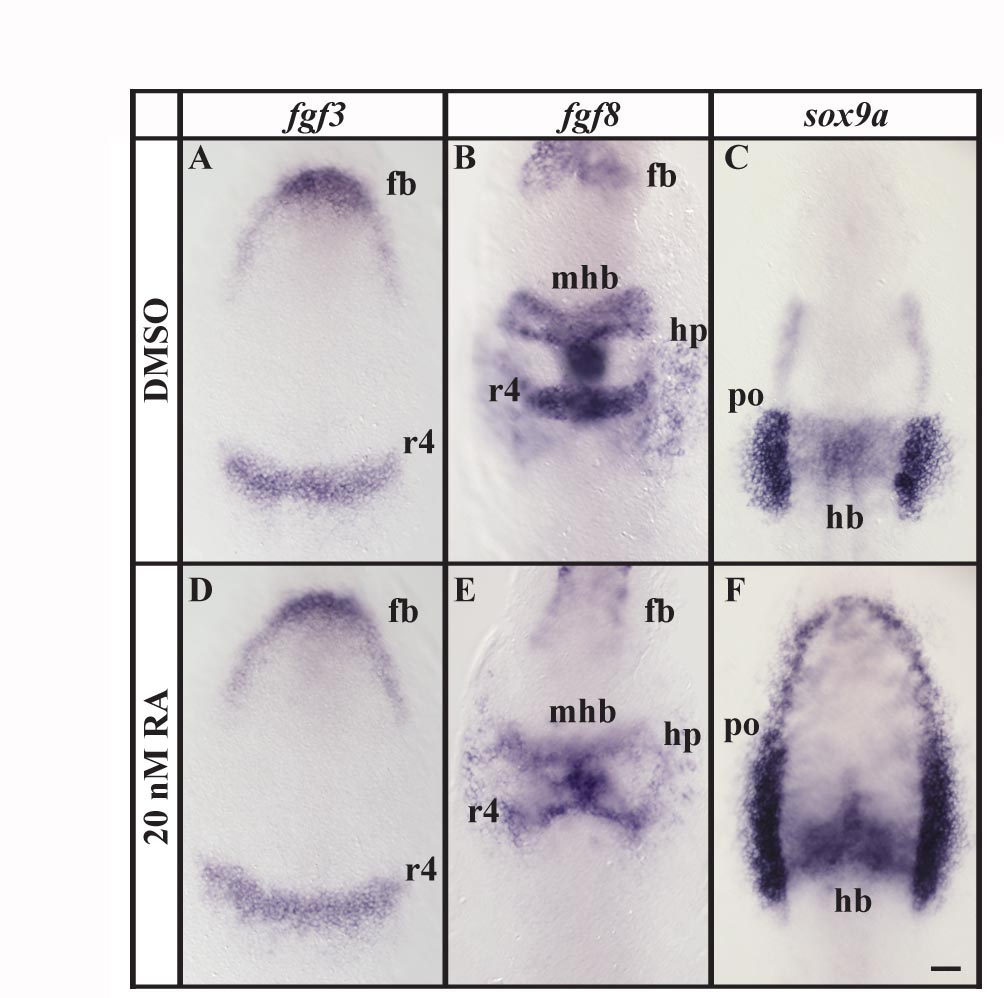

Supplement: Additional file 2 — Retinoic acid treatment has little effect on patterning along the anterior-posterior axis. (A, B, D, E) Expression of fgf3 or fgf8 in embryos treated with 20 nM RA is indistinguishable from control embryos treated with DMSO. (C, F) In RA treated embryos expression of sox9a in the preotic region expands to surround the anterior neural plate border in comparison to control embryos. However, sox9a expression in the hindbrain is identical in RA and DMSO treated embryos. Dorsal views of 1–5-somite stage embryos with anterior towards the top. fb, forebrain; hb, hindbrain; hp, heart primordium; mhb, midbrain-hindbrain border; r4, rhombomere 4; po, preotic region. Scale bar: 40 μm μm. [file 1471-213X-7-5-S2.jpeg]

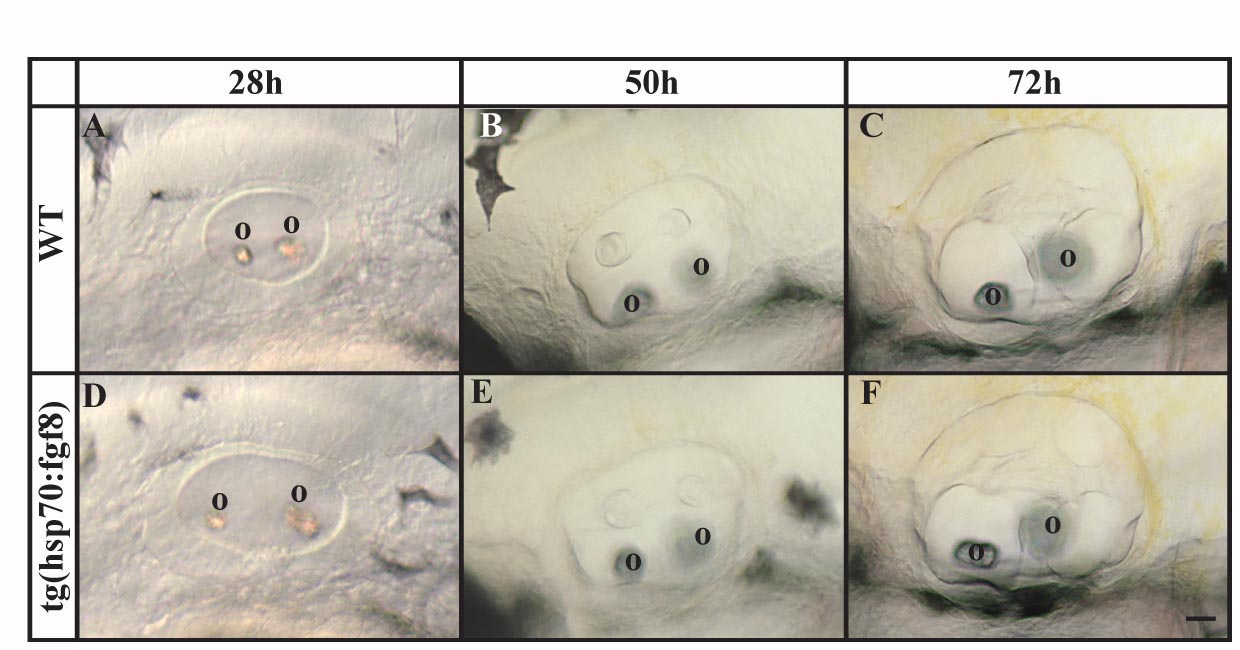

Supplement: Additional file 3 — The increase in otic tissue, following a heat shock at late gastrulation stages of transgenic hsp:fgf8 embryos, is transient. (A, D) At 28 hours post fertilization, otic vesicles in transgenic fish heat shocked at late gastrulation stages are still larger than in non-transgenic siblings. (B, C, E, F) The size difference of otic vesicles in transgenic and non-transgenic embryos is less prominent at 50 hours post fertilization, and indistinguishable by 72 hours post fertilization. Lateral views of live otic vesicles with anterior to the left and dorsal towards the top. o, otolith. Scale bar: 120 μm. [file 1471-213X-7-5-S3.jpeg]
